# Male Microchimerism at High Levels in Peripheral Blood Mononuclear Cells from Women with End Stage Renal Disease before Kidney Transplantation

**DOI:** 10.1371/journal.pone.0032248

**Published:** 2012-03-05

**Authors:** Laetitia Albano, Justyna M. Rak, Doua F. Azzouz, Elisabeth Cassuto-Viguier, Jean Gugenheim, Nathalie C. Lambert

**Affiliations:** 1 UMC Transplantation Rénale, Hôpital Pasteur, Centre Hospitalo-Universitaire de Nice, Nice, France; 2 INSERM UMR1097, Parc Scientifique de Luminy, Marseille, France; 3 Service de Chirurgie et Transplantation Hépatique, Hôpital l'Archet 2, Nice, France; 4 Université de Nice Sophia Antipolis, Nice, France; 5 INSERM U526, IFR 50, Faculté de Médecine, Université de Nice Sophia Antipolis, Nice, France; VU University Medical Center, Netherlands

## Abstract

Patients with end stage renal diseases (ESRD) are generally tested for donor chimerism after kidney transplantation for tolerance mechanism purposes. But, to our knowledge, no data are available on natural and/or iatrogenic microchimerism (Mc), deriving from pregnancy and/or blood transfusion, acquired prior to transplantation. In this context, we tested the prevalence of male Mc using a real time PCR assay for DYS14, a Y-chromosome specific sequence, in peripheral blood mononuclear cells (PBMC) from 55 women with ESRD, prior to their first kidney transplantation, and compared them with results from 82 healthy women. Male Mc was also quantified in 5 native kidney biopsies obtained two to four years prior to blood testing and in PBMC from 8 women collected after female kidney transplantation, several years after the initial blood testing. Women with ESRD showed statistically higher frequencies (62%) and quantities (98 genome equivalent cells per million of host cells, gEq/M) of male Mc in their PBMC than healthy women (16% and 0.3 gEq/M, p<0.00001 and p = 0.0005 respectively). Male Mc was increased in women with ESRD whether they had or not a history of male pregnancy and/or of blood transfusion. Three out of five renal biopsies obtained a few years prior to the blood test also contained Mc, but no correlation could be established between earlier Mc in a kidney and later presence in PBMC. Finally, several years after female kidney transplantation, male Mc was totally cleared from PBMC in all women tested but one. This intriguing and striking initial result of natural and iatrogenic male Mc persistence in peripheral blood from women with ESRD raises several hypotheses for the possible role of these cells in renal diseases. Further studies are needed to elucidate mechanisms of recruitment and persistence of Mc in women with ESRD.

## Introduction

Microchimerism (Mc) is the presence of a small amount of foreign cells or DNA within a person's circulation or tissues [1]. Mc can be acquired through iatrogenic interventions such as organ transplantation, first described in liver transplantation in 1969 [2], or blood transfusion [3]. Mc can also be naturally acquired during pregnancy due to feto-maternal traffic of cells through the placenta membrane [4]. Interestingly, these cells are not short term transitory cells as they can persist for decades in small quantities in their respective hosts [5]. Exchange of cells between fetuses can also contribute to natural Mc within an individual. They were first described between bovine dizygotic twins [6] and later in humans [7]. Recently, our group even reported the presence of cells from an unrecognized (vanished) twin in a 40-year-old man diagnosed with a scleroderma-like disease [8].

The natural phenomenon of Mc has already been investigated in whole peripheral blood [9], peripheral blood mononuclear cells (PBMC) [10] and different tissues [11] from healthy women and women with autoimmune diseases as scleroderma, dermatomyositis, thyroiditis [12], [13], [14], [15]… Higher quantities and frequencies of male Mc observed in women with scleroderma compared to matched controls suggested a possible role for these cells in autoimmunity [12]. However it is still unclear whether the presence of Mc is the cause or the consequence of autoimmunity, whether natural Mc is present to heal or to kill (for reviews [16], [17]). For example, in breast cancer, Mc was seen as a protective factor in a study by Gadi et al., where the risk of cancer was lower in women positive for male Mc at the peripheral level [18], whereas in another study, on human breast carcinoma developing during pregnancy, presence of fetal Mc in tumor sections suggested these cells played a detrimental role [19].

Evaluation of the role of fetal Mc in the context of renal diseases was mostly studied indirectly. Indeed fetal cells have been found twice as often in kidneys from women with systemic lupus erythematosus (SLE) than in normal kidneys [20], suggesting that they could play a role in renal disease and/or renal function. A prior study in patients with SLE noted a higher mean number of male equivalent cells in peripheral blood from patients with renal disease than from patients with no renal involvement (4.2 male equivalent cells vs 0.89 male equivalent cells respectively; p<0.05) [21].

When chimerism is studied in patients with renal diseases it is generally to analyze the influence of donor Mc after kidney transplantation for tolerance mechanism purposes [22], and not to analyze the potential role and fate of natural and/or iatrogenic Mc acquired prior to transplantation.

In this context, we studied the unexplored phenomenon of Mc in women with end stage renal diseases (ESRD) prior to their first kidney transplantation, by using a quantitative PCR method for male Mc detection in their PBMC. Male Mc quantification was estimated according to the source of chimerism, pregnancy or transfusion, and compared to results obtained from healthy women.

## Methods

### Participant' characteristics

Fifty-five women awaiting their first kidney transplantation and 82 healthy women were studied. Controls and patients came from the same geographical area between Marseille and Nice, in the south east of France.

All 55 women with chronic kidney disease were hemodialyzed except for 4 with a Cockroft and Gault creatinine clearance <15 ml/min at the time of DNA extraction. The initial nephropathies were interstitial (n = 21), glomerulonephritis (n = 11), polycystic kidney disease (n = 9), nephroangiosclerosis (n = 4), lupus nephritis (n = 3), diabetes type I (n = 2), diabetes type 2 (n = 2), hemolytic-uremic syndrome (n = 2), indeterminate (n = 1). This proportion is similar to those described in transplanted patients by the French ESRD Registry REIN 2009.

Women with ESRD and healthy women were very similar for pregnancy history and differed for transfusion history (leuko-reduced) as detailed in **Table 1**.

**Table 1:** Characteristics from healthy women and women with ESRD.

Characteristics	Women with ESRD (N = 55)	Healthy women (N = 82)	P values
Median age, range	50[14–67]	52 [37–69]	ns
Mean number of children	2	2 (N = 81)^a^	ns
Mean number of sons	1	1(N = 81)	ns
% of women with at least one son	69	62 (N = 81)	ns
Mean age of the youngest son	19	21 (N = 81)	ns
% of nulligravid women	10	6 (N = 81)	ns
% of women with early pregnancy loss	43	53 (N = 81)	ns
% of women with blood transfusion	65	12	<0.0001
Mean number of transfusions	1	0	<0.0001
Years since last transfusion: mean, [range]	5.6 [0.5–30]	24.5 [17]–[36]	<0.0001

a pregnancy and transfusion information was incomplete for one healthy woman. ns: not significant.

### Ethics Statements

All controls were healthy women with no history of autoimmune disease or kidney disease. These healthy women have been used as controls in a previous published study [23]. This study received the approval from the French Ethical Committee Marseille 2 and is registered at the INSERM under the Biomedical Research Protocol number RBM-04-10. Written consent forms obtained according to the Declaration of Helsinki [24] were signed. Questionnaires with detailed information about previous transfusions, pregnancies, and existence of an older brother (as a possible source of male Mc) were filled in for each participant of the study. For one healthy control, we were not able to obtain all the information. Samples from women with ESRD were collected for HLA-typing before registration on the waiting list and then for microchimerism detection which was performed as a “res nullus” analysis. Patients were informed and acquiescent.

### DNA extraction from PBMC and native kidney parenchyma

DNA extractions from PBMC for women with ESRD were performed prior to the extractions from controls obtained for a different study. Genomic DNA from patients was extracted using a “salting-out” method [25], from PBMC after EDTA blood processing by Ficoll Histopaque 1077 gradient centrifugation (Sigma-Aldrich, St Louis, MO, USA). Genomic DNA was quantified and purity was assessed by spectrophotometric absorbance at 260 and 280 nm.

Genomic DNA from controls was extracted from PBMC after EDTA blood processing by Ficoll Histopaque 1077 gradient centrifugation (Sigma-Aldrich, St Louis, MO, USA). DNA isolation was done with an EZ1 DNA Tissue Kit (Qiagen, Hilden, Germany) on a BIOROBOT® *EZ1* according to the manufacturer's instructions.

Aware that different DNA extraction methods between the two groups could lead to different results for Mc, 9 patients with ESRD with blood taken more recently had their DNA extracted with a similar method to healthy women (Qiagen kit) and were tested as a separate group to verify whether different methods lead to different results (see results).

For 5 patients, renal tissues from native kidneys were obtained by transcutaneous biopsy and cryopreserved. DNA was extracted with an EZ1 DNA Tissue kit (Qiagen, Hilden, Germany) as described above.

### DYS14 real time quantitative PCR

Quantification of male Mc was obtained by real-time PCR for a Y-chromosome specific sequence DYS14 on a Light Cycler® with Light Cycler® Fast Start DNA MasterPLUS Reaction kits (Roche, Indianapolis, IN, USA) as previously described [23]. Total amount of tested DNA was measured by ß-globin, a house keeping gene, as previously described [13]. Duplicates of ß-globin were averaged for each woman, giving the total number of cell equivalents multiplied by the number of wells tested.

Sensitivity of the DYS14 assay was accurate to the equivalent DNA of 1 male cell in a background equivalent DNA of 20,000 female cells. DNA from each participant was tested in ten samples with a DNA equivalent of 20,000 cells by real-time PCR for ß-globin (equivalent of 200,000 cells tested/woman). For ease of result legibility, the amount of male DNA was expressed as the number of genome equivalent male cells per million female cells (gEq/M). Because the male Mc in each well is assumed to have a Poisson distribution, the male genome equivalent cells for each subject were averaged as previously described [23]. The estimate for individuals for whom all replicates are assayed using the same number of cells per replicate is the usual Poisson estimate: *−ln (1−p)/M* where *p* is the fraction of samples with at least 1 male cell (the limit of p being 1 well positive out of 10 tested) and M is the number of cells in the sample (20,000 in our case). The confidence limit for calculation is when 1 well is positive out of 10, this is why as a conservative estimate of the quantity of male DNA, we required that a sample had at least two wells out of ten positive. Extreme caution was employed to avoid PCR contamination: women performed all the technical work. Pre-amplification steps were carried out in a separate room. A negative control sample was included in each experiment.

### Statistical analysis

Significant differences between the two groups were detected using the Chi square test for qualitative variables (Fisher exact test) and an unpaired t-test for continuous variables with a normal distribution or if not, a non-parametric test i.e a Wilcoxon signed-rank test and a Mann-Whitney test for paired series using Statview 5 software (SAS Institute Inc;Cary, NC; USA). Logistic regression was used to assess a relationship between renal status and the presence of male DNA. For all tests, statistical significance was defined as p<0.05.

## Results

### Increased frequency and higher quantities of male Mc in PBMC from women with ESRD compared to controls

**Figure 1:**
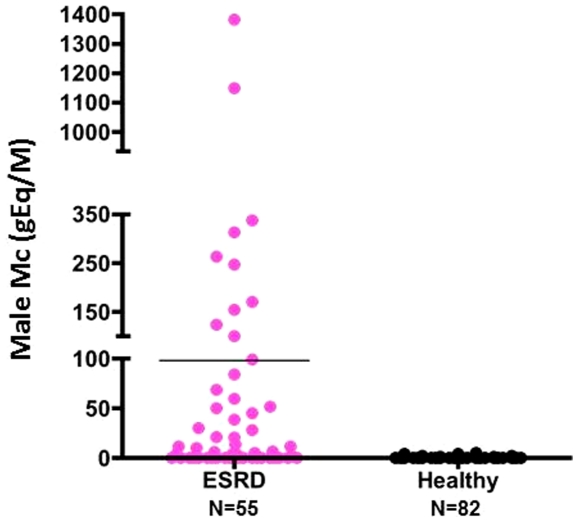
Male Mc quantities in PBMC from women with ESRD and healthy women.

**Figure 2:**
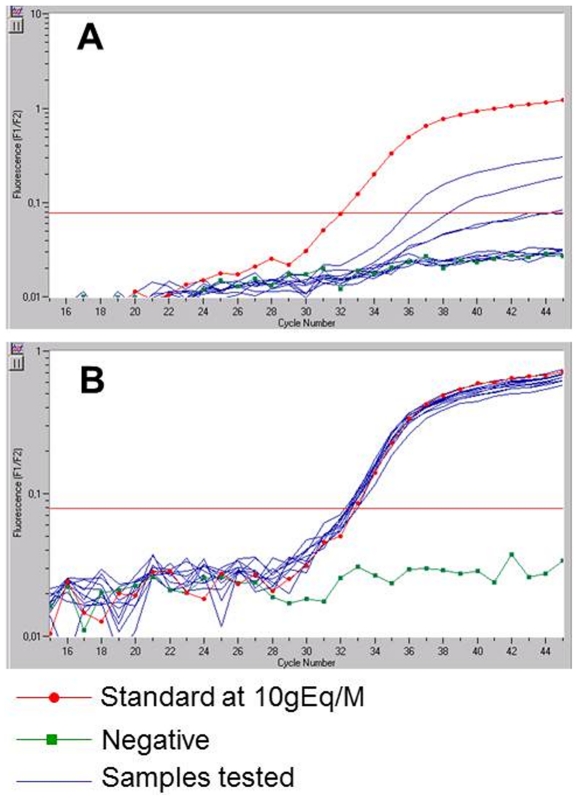
Typical amplifications of male Mc in female host's DNA. PBMC from a healthy woman (A) and a woman with ESRD (B) tested for male Mc in 10 samples.

When both groups were analyzed as a whole, without stratifying by age, parity, or history of transfusion, as illustrated in **Figure 1**, we detected male Mc in 62% (34/55) of women with ESRD versus only 16% (13/82) of healthy women (p<0.00001). Differences in quantities of Mc between the two groups were also very significant (**Figure 1**, **Figure 2** for typical amplifications and **Table S1** for number of wells positive). Levels of Mc ranged from 0 to 1382 gEq/M in women with ESRD and from 0 to 5 gEq/M in controls with a mean number of 98 gEq/M and 0.3 gEq/M respectively (p = 0.0005). We did not find any correlation between Mc levels and the length of time since the last abortion, the number of induced or spontaneous abortions, the length of time since the last transfusion, the length of time since the birth of the last child or last son, or the number of sons or children (data not shown).There was also no relationship between the types of nephropathy (vascular, glomerular, interstitial and polycystic) and the presence or the level of Mc (data not shown).

As differences in DNA extraction methods between women with ESRD and healthy women could introduce artifacts for Mc results, we analyzed a separate subgroup of 9 patients with ESRD with blood taken more recently, for whom DNA extraction methods were identical to healthy controls. Frequency of women positive for Mc was statistically significant in this subgroup of patients with ESRD (Fisher's exact test, p = 0.013), with 5 out of 9 women with ESRD positive compared to 13 out of 82 healthy women. Levels of Mc were also significantly increased (Mann Whitney, p = 0.0006) with respectively a mean of 57.0 gEq/M [95% CI: −12.46–126.6] and 0.3 gEq/M [95%.CI: 0.11–0.54]. Results of this sub-analysis exclude a possible bias due to different DNA extraction methods.

### Frequencies and quantities of Mc are increased in women with ESRD whether they had or not a history of male pregnancy and/or of transfusion

Male Mc could come from natural or iatrogenic source; we therefore classified women with ESRD and healthy women according to whether they had or not a transfusion history and they had or not given birth to at least one son (**Table 2**). Non-transfused (TSF−) women with ESRD had male Mc more often than healthy matched controls, regardless of whether they had given birth to a son or not (S+ or S−), with respectively 50% and 71% compared with 18% and 14% (p = 0.02 and p = 0.001 respectively). Moreover quantities of male Mc were higher in non-transfused women with ESRD compared to matched healthy women and again differences were not due to pregnancy history as results were similarly significant whether they had or not given birth to at least one son (p = 0.0019 and <0.0001 respectively).

**Table 2:** Male Mc in women with ESRD and healthy women according to pregnancy and transfusion history.

Analyzed group		Women positive for Mc # (%)	Frequency p-values	Mean quantity of male Mc (gEq/M) [range]; Median	Quantity p-values
**TSF−S+**	Controls (N = 44)	8 (18%)		0.3 [0–5]; 0	
	ESRD (N = 12)	6 (50%)	0.05	36.5 [0–247]; 2	0.0019
**TSF−S−**	Controls (N = 28)	4 (14%)		0.4 [0–4]; 0	
	ESRD (N = 7)	5 (71%)	0.006	37.4 [0–101]; 28	<0.0001
**TSF+S+**	Controls (N = 7)	0 (0%)		0	
	ESRD (N = 26)	17 (65%)	0.003	130 [0–1149]; 16.5	0.008
**TSF+S−**	Controls (N = 3)	1 (33%)		0.6 [0–2]; 0	
	ESRD (N = 10)	6 (60%)	No stats *	192.2 [0–1382]; 4.5	No stats*

TSF+: women who had received at least one blood transfusion; TSF−: women who had never received a blood transfusion; S+: women who had given birth to at least one son; S−: women who had never given birth to a son (S−);

* no stats: statistical analyses were not done due to small numbers.

Similarly, transfused (TSF+) women with ESRD also had male Mc more often and in higher quantities than healthy matched controls whether they had given birth to a son, with only the former case (S+) that could be statistically evaluated (p = 0.008) due to small numbers in the latter case (S−).

### Male Mc in kidney biopsies two to four years prior to blood testing

Five women were investigated for male DNA in their kidney parenchyma (**Table 3**). Renal tissues were obtained from kidney biopsies with a median time of 36 months (range from 24 to 48) prior to blood testing. Among the five patients, 1 had anti-neutrophil cytoplasmic antibodies (ANCA), 2 had systemic lupus erythematosus (SLE), 1 hemolytic-uremic syndrome (HUS), and 1 focal glomerulosclerosis (FSGS). Two of the five patients were negative for male Mc in renal parenchyma and a few years later had either 10 gEq/M in their PBMC or no male Mc. Three women carried male DNA at concentrations of 30, 10 and 3 gEq/M in renal tissue and a few years later had respectively 21, 2 and 474 gEq/M in their PBMC. No correlation was found between earlier levels in kidney biopsies and later levels of Mc in PBMC.

**Table 3:** Quantification of Mc in kidney biopsies prior to transplantation from five women with ESRD.

Patients	Kidney disease	Results of male Mc in kidneys (gEq/M)	Year of kidney biopsy	Results of male Mc in PBMC (gEq/M)	Year of blood test	Months between kidney biopsy and blood test
1	ANCA*	**30**	1999	**21**	2002	36
2	SLE	0	2003	**10**	2005	24
3	SLE	0	1999	0	2002	36
4	HUS	**10**	2000	**2**	2004	48
5	FSGS	**3**	2000	**474**	2002	36

* ANCA: antineutrophil cytoplasmic antibodies, SLE: systemic lupus erythematosus; HUS: hemolytic-uremic syndrome, FSGS: Focal segmental glomerulosclerosis.

### Clearance of male Mc after kidney transplantation in PBMC from 8 women with ESRD (Table 4)

**Table 4:** Quantification of male Mc in PBMC from 8 women with ESRD after female kidney transplantation.

Patients	Male Mc in PBMC (gEq/M)		Years after transplantation	GFR* (ml/min) at the time of post-transplant blood test
	before transplantation	after transplantation		
1	**1149**	0	6	67
2	0	0	5	53
3	**14**	0	6	60
4	**21**	**6**	5	49
5	0	0	3	25
6	**45**	0	7	59
7	0	0	4	44
8	**5**	0	5	32

* GFR: glomerular filtration rate.

All patients from this table are different from patients presented table 3, except Patient 4 who is Patient 1 in Table 3.

Finally, we quantified male Mc in PBMC from 8 women with ESRD who had received a female kidney transplant (so as not to complicate Mc sources). At the time of DNA extraction from PBMC, all 8 kidney grafts were functional with a glomerular filtration rate (GFR) ranged from 25 to 70 ml/min. The immunosuppressive protocol consisted in an induction by anti-lymphocyte serum in all patients and triple drugs regimen (steroids, calcineurin inhibitors and mycophenolic acid) therapy. PBMC analysis was carried out in a median time of 4.6 years after transplantation. Among the 8 women tested, 3, negative in their PBMC prior to transplantation remained negative after transplantation, 5 positive before transplantation with, from the lowest to the highest results: 5, 14, 21, 45 and 1,149 gEq/M were all negative after transplantation except the third patient who had 6 gEq/M in her PBMC. Using a Wilcoxon signed-rank test, which is a non-parametric test, we found a marginal decrease (p = 0.04) of Mc levels in the pre to post transplantation period.

## Discussion

We present the first study analyzing male Mc in PBMC from women with end stage renal disease (ESRD), prior to their first kidney transplantation. Male Mc was found significantly more often and at higher concentrations than in healthy women. Samples were collected from two independent studies and DNA extractions obtained by different methods, which could introduce artefacts in the results. Noteworthy, divergences between methods have been demonstrated with *circulating DNA* from plasma or urine samples, where small DNA fragments were lost [26] depending on DNA extraction methods but not from cell DNA samples. However to eliminate any suspicion, we tested independently a subgroup of 9 patients for whom DNA was extracted with a similar method to healthy women and found similar results to those obtained in the main group of women with ESRD.

Women with ESRD often have a history of leuko-reduced blood transfusion which could leave iatrogenic Mc as a post transfusion consequence and trigger higher levels of male Mc, when the donor was male [3]. However, we demonstrated that the difference for Mc frequency and/or quantity observed in women with ESRD was not dependent on transfusion history, as results remained significant in women who had never had a blood transfusion. Intriguingly, the difference observed did not correlate either with pregnancy history, since having given birth to a son or not had no influence on the results. Furthermore, women with ESRD who had never had a blood transfusion and never given birth to a son had male Mc more often in their PBMC and at higher quantities than healthy matched controls. These surprising results suggest they have male DNA from an incomplete pregnancy and/or an unrecognized twin as previously discussed in other studies relative to Mc [27], [28]. Indeed, a non-negligible number of pregnancies end before they are clinically noticed [29] and unrecognized twinning is relatively common in healthy pregnancy [30]. In a recent study, Kremer Hovinga et al., also suspected such non-classical sources as principal causes for Mc in renal biopsies from women with lupus nephritis as they showed no significant difference between the occurrence of chimerism in the biopsies of women who had been pregnant compared with women who had not been pregnant [31].

Several hypotheses, not necessarily exclusive, could explain the higher prevalence of male Mc in women with ESRD before kidney transplant compared to healthy women.

First, high levels of male Mc observed in peripheral blood mononuclear cells could indicate a decreased capacity to eliminate male DNA, a consequence of ESRD by decrease of glomerular filtration rate. Very little is known about the life cycle, persistence and elimination process of foreign or semi-foreign cells within an individual. It has been shown that fetal DNA disappears from peripheral blood right after delivery in a very rapid, probably immunological and/or renal, process [32]. Moreover, male DNA has been found in female urine in several cases: after male kidney transplantation and during pregnancy with male fetuses [33]. However, it is still unknown how DNA crosses the normal kidney barrier and appears in the filtrate. Our initial results, on peripheral blood from 8 women who had received a female kidney transplant more than 4 years before, seem to argue in favour of a recovered capacity to eliminate male Mc after transplantation. Indeed women with ESRD who were previously positive for male Mc became negative after kidney transplant. However it is still speculative to consider that clearance of male DNA observed post-transplant in PBMC is due to restored kidney function, as patients undergoing kidney transplant are under strong immunosuppressive drugs that could also affect microchimerism levels. Illustrating this, we recently found, in women with Rheumatoid Arthritis, fluctuating levels of Mc coinciding with disease flare up and treatment [34].

A second hypothesis for high levels of Mc in blood could be a consequence of inflammation in dialysis as well as ESRD patients. It has been recognized that 30% to 50% of pre-dialysis, hemodialysis and peritoneal dialysis patients have serological evidence of an activated inflammatory response [35]. Many mechanisms can induce an inflammatory condition such as reduced renal clearance of cytokines, accumulation of advanced glycation end-products (AGEs), chronic heart failure, atherosclerosis *per se*, unrecognized persistent infections with additional causes in dialysis such as fistula infection and bioincompatibility of dialysis membrane, exposure to endotoxins [35]. High levels of cytokines and chemoattractants may possibly recruit fetal cells from their niche, for example from bone marrow or lymph nodes [36], [37]. Here again the quasi-absence of Mc in PBMC after kidney transplantation could come from decreased inflammation due to strong immuno-suppression targeting T lymphocytes.

Thirdly, microchimeric cells could be mobilized to repair damaged tissue and high blood levels would only be reflecting higher kidney levels. In a rat model, it has been described that fetal cells could remodel the maternal kidney after injury [38]. Therefore, the presence of male Mc in kidneys, although at low levels, could be a regenerative process. In our study, we were able to obtain 5 native kidneys biopsies taken 24 to 48 months prior to the blood test, at the time of diagnosis, and demonstrate that 3 out of 5 tested were slightly positive for Mc, but we could not demonstrate whether these cells belonged to the kidney or came from blood vessels supplying the organ. This would have to be determined in further analyses and was beyond the scope of the current study.

Finally as a fourth hypothesis, fetal cells recruited under inflammatory processes might not be bystanders in peripheral blood, or helpers, but effector cells as suggested in a recent study on kidney biopsies from patients with lupus nephritis. In these biopsies microchimeric cells were indeed shown within the hematopoietic stem cell phenotype (CD34+) as well as within T lymphocytes [20].

As anticipated, there is no single explanation for our results showing high levels of male Mc in women with ESRD. Even if we speculate that Mc cells are recruited to help the damaged organ, it is obvious that this help is not fully efficient, since the kidney is not functional in the end. However, it is to be noted that a few studies describe slower disease progression in women with chronic renal disease compared to men, which could argue for a protective role of Mc. Indeed, a meta-analysis involving 11,345 patients determined a gender effect on kidney disease progression from non-diabetic patients [39]. Men with autosomal dominant polycystic kidney disease, membranous nephropathy, or chronic kidney disease of unspecified etiology progressed to renal failure more rapidly than women. Female protection from renal disease progression is also observed in animal models of progressive renal disease [40]. Little is known about the mechanisms underlying sex differences in renal disease susceptibility [41] and it would be interesting to investigate whether chimerism could contribute to a certain “protection” and lead to a gender difference.

This article brings forward new original insights into the unexplored phenomenon of Mc in renal diseases and should initiate future research to determine mechanisms of recruitment and persistence of Mc in patients with ESRD prior to kidney transplantation.

## Supporting Information

Table S1 **Distribution of male Mc per 10 wells tested among positive individuals.** Mean number of positive wells out of ten tested in women with ESRD: 5.85 (95% CI [4.928–6.778]) and in healthy women: 2.77 (95% CI [1.274–4.265]). The difference is statistically significant: p = 0.0016, Mann-Whitney test.
(DOCX)
