# Supplementary material for: Male Microchimerism at High Levels in Peripheral Blood Mononuclear Cells from Women with End Stage Renal Disease before Kidney Transplantation
Source: PLoS One. 2012 Mar 5;7(3):e32248. doi: 10.1371/journal.pone.0032248 (PMC3293902; doi:10.1371/journal.pone.0032248)
Supplement: Table S1 — Distribution of male Mc per 10 wells tested among positive individuals. Mean number of positive wells out of ten tested in women with ESRD: 5.85 (95% CI [4.928–6.778]) and in healthy women: 2.77 (95% CI [1.274–4.265]). The difference is statistically significant: p = 0.0016, Mann-Whitney test. (DOCX) [file pone.0032248.s001.docx]

| **ESRD women positive for Mc** | **Number of positive wells out of 10 tested** | **Healthy women positive for Mc** | **Number of positive wells out of 10 tested** |
| --- | --- | --- | --- |
| **CKD1** | **2** | **NRL-001A** | **2** |
| **CKD2** | **2** | **NRL-002A** | **5** |
| **CKD3** | **9** | **NRL-003A** | **2** |
| **CKD5** | **8** | **NRL-031A** | **2** |
| **CKD6** | **6** | **NRL-035A** | **2** |
| **CKD7** | **2** | **NRL-049A** | **4** |
| **CKD8** | **3** | **NRL-050A** | **4** |
| **CKD9** | **3** | **NRL-052A** | **3** |
| **CKD10** | **6** | **NRL-053A** | **2** |
| **CKD11** | **10** | **NRL-056A** | **3** |
| **CKD12** | **10** | **NRL-057A** | **3** |
| **CKD13** | **4** | **NRL-112A** | **2** |
| **CKD14** | **10** | **NRL-117A** | **2** |
| **CKD15** | **7** |  |  |
| **CKD17** | **10** |  |  |
| **CKD19** | **5** |  |  |
| **CKD20** | **6** |  |  |
| **CKD21** | **8** |  |  |
| **CKD23** | **9** |  |  |
| **CKD26** | **10** |  |  |
| **CKD30** | **9** |  |  |
| **CKD32** | **2** |  |  |
| **CKD34** | **3** |  |  |
| **CKD35** | **5** |  |  |
| **CKD37** | **2** |  |  |
| **CKD38** | **10** |  |  |
| **CKD39** | **2** |  |  |
| **CKD44** | **2** |  |  |
| **CKD48** | **3** |  |  |
| **CKD49** | **6** |  |  |
| **CKD51** | **8** |  |  |
| **CKD52** | **8** |  |  |
| **CKD54** | **2** |  |  |
| **CKD55** | **7** |  |  |
